# Pragmatic spatial sampling for wearable MEG arrays

**DOI:** 10.1038/s41598-020-77589-8

**Published:** 2020-12-10

**Authors:** Tim M. Tierney, Stephanie Mellor, George C. O’Neill, Niall Holmes, Elena Boto, Gillian Roberts, Ryan M. Hill, James Leggett, Richard Bowtell, Matthew J. Brookes, Gareth R. Barnes

**Affiliations:** 1 Wellcome Centre for Human Neuroimaging, UCL Institute of Neurology, London, WC1N 3AR UK; 2 Sir Peter Mansfield Imaging Centre, School of Physics and Astronomy, University of Nottingham, University Park, Nottingham, NG7 2RD UK

**Keywords:** Magnetoencephalography, Image processing

## Abstract

Several new technologies have emerged promising new Magnetoencephalography (MEG) systems in which the sensors can be placed close to the scalp. One such technology, Optically Pumped MEG (OP-MEG) allows for a scalp mounted system that provides measurements within millimetres of the scalp surface. A question that arises in developing on-scalp systems is: how many sensors are necessary to achieve adequate performance/spatial discrimination? There are many factors to consider in answering this question such as the signal to noise ratio (SNR), the locations and depths of the sources, density of spatial sampling, sensor gain errors (due to interference, subject movement, cross-talk, etc.) and, of course, the desired spatial discrimination. In this paper, we provide simulations which show the impact these factors have on designing sensor arrays for wearable MEG. While OP-MEG has the potential to provide high information content at dense spatial samplings, we find that adequate spatial discrimination of sources (< 1 cm) can be achieved with relatively few sensors (< 100) at coarse spatial samplings (~ 30 mm) at high SNR. After this point approximately 50 more sensors are required for every 1 mm improvement in spatial discrimination. Comparable discrimination for traditional cryogenic systems require more channels by these same metrics. We also show that sensor gain errors have the greatest impact on discrimination between deep sources at high SNR. Finally, we also examine the limitation that aliasing due to undersampling has on the effective SNR of on-scalp sensors.

## Introduction

Magnetoencephalography (MEG) has become a vital tool for studying human brain function in both basic and clinical settings^1–4^. Simulation studies show that MEG system performance continues to improve with an increase in channel count as long as channel noise is uncorrelated^5^. There is however a balance between system cost and channel redundancy. Based on work over the last 20 years^5–8^, the design of whole-head Superconducting Quantum Interference Device (SQUID) based systems stabilized to around 300 channels. While source localization performance has a strong dependence on channel count^5^, there is a complex interplay between the methods used to invert the lead-field matrix, selection of a forward model, sampling density, and the geometry of the source space^6,9–15^.

These issues have come back into focus with emergence of new MEG system technologies. These include the advent of high critical temperature SQUIDs (high T_c_-SQUIDS), nitrogen vacancy magnetometers and optically pumped magnetometers (OPMs)^16–21^. These new sensors allow for the construction of MEG arrays that can be brought closer to the scalp (within a few mm) and in the case of OPMs, offer the flexibility to image human brain function during subject movement^22–24^ ( with the crucial addition of field nulling coils^25–27^). From here on we refer to these methods collectively as on-scalp MEG as opposed to typical helium-based cryogenic systems, which are often displaced some distance (~ 20 mm) from the scalp (off-scalp system).

As on-scalp sensors are a typically placed much closer to the scalp than off-scalp sensors, they measure a three to fivefold increase in signal^15,28^. In principle, this should offer the capability of resolving neuronal activity with a higher spatial discrimination as smaller spatial wavelengths can potentially be sampled^6,29^. Indeed recent work^30^ has shown that on-scalp systems would benefit from up to 300 spatial samples in order capture these small spatial wavelengths. An important point to note is that sparser arrays could result in under-sampling (of the high spatial frequency features) and this will give rise to unexplained noise due to signal aliasing.

Here we argue that for a wide variety of neuroscience applications, maximising spatial discrimination, although desirable, is not absolutely necessary. For instance, in paediatric epilepsy surgery, a crucial clinical application of MEG^31–36^ a large percentage of brain volume (~ 5–10%, equivalent to a 2–3 cm radius sphere) may be resected^37^. The clinical question does not require the spatial discrimination between sources at a millimetre scale. In the case of basic neuroscience applications that average results across subjects their spatial discrimination is limited by the functional and anatomical variability that exists between subjects^38–41^. Furthermore, in studies which investigate electrophysiological functional connectomes, the spatial discrimination required may be on the scale of 1–2 cm, as a single time course representing an entire atlas-defined parcel is often utilised^42^. Rather than strive to maximize spatial discrimination or information content, an alternative question is “given a desired discrimination what is the sampling density/ sensor number I require?” We approach the question of discrimination in a statistical framework by asking “at which sensor density one is able to confidently (p < 0.05) distinguish between two competing source models”^43–45^. By adopting this approach we can design MEG arrays around a given scientific question (or spatial discrimination criterion) and therefore minimize channel count. We note that the answer to this question will be conditional on Signal to Noise Ratio (SNR), which itself will be a function of any aliasing observed due to undersampling.

In this work we also consider the issue of sensor calibration. Unlike SQUID based systems, where the feedback electronics maintains a constant linear relationship to applied flux, optically pumped magnetometers are particularly vulnerable to external factors which give rise to a change in their gain. Perhaps the most pernicious of these errors is due to the inherent nonlinear response of the sensor^21^. When operating in the spin exchange relaxation Free (SERF) regime these sensors are only linear within a few nT of zero field^22^. These gain errors can be caused by subject movement through a non-zero background field or by the change in the ambient magnetic field over time^26,27^. Some form of active shielding^27,46^ or closed-loop feedback would typically be required to minimize these issues. Another issue is how the sensors interact with each other in multichannel systems. On-board coils that produce magnetic fields for field zeroing or amplitude modulation^20,47^ may change the gain of a nearby sensor. This problem is static (if the sensors do not move relative to one another) and deterministic but assumes that a suitable calibration procedure is in place to correct for these issues^48^. Additionally the use of custom designed on-board coils can reduce the impact of this issue^49^.

The paper proceeds as follows. First we outline the model comparison framework and the competing models used to define spatial discrimination. We examine these discrimination estimates for different sensor numbers under different SNR conditions for deep and shallow dipolar sources using on-scalp sensors. We use the same methods to investigate cryogenic sensors layouts and show, in accord with previous studies^50^, that comparable discrimination for on-scalp sensors is achievable with fewer sensors. We also examine the impact of sensor gain error^21,22,26^ on discrimination. Finally, we consider the effects of aliasing due to undersampling.

## Methods

### Simulating MEG data

All simulations and source reconstructions are based on fields generated by single dipolar sources. We simulate on-scalp MEG data based on sensors offset by 6.5 mm from the scalp. For comparison, we also simulate off-scalp MEG with identical simulation parameters except that the offset from the scalp is 20 mm. Each dipole source was given a 10 Hz sinusoidal profile and recorded for a single epoch of 1000 ms. The sensor level SNR of the response was manipulated by changing the source amplitude from 1 to 10 nAm to 100 nAm assuming a 100 fT noise standard deviation (uncorrelated across sensors) in all cases (corresponding to a $10\mathrm{ fT}/\sqrt{\mathrm{Hz}}$ noise floor in 100 Hz bandwidth).

The mesh used to generate these datasets was the MNI canonical mesh available in SPM12. The separation between vertices is approximately 3 mm on average. The orientation of the source is defined by the surface normal of the cortical mesh at that location. The forward model was the Nolte single shell model^51^ and sensors were assumed to be point magnetometers. A total of 50 different array designs were considered with inter sensor spacing varying between 10 and 60 mm (the method of array design is covered in “Array design” section).

For each of the 50 array designs, 40 different datasets were generated. Each dataset contained data from a different single dipolar source with added noise. The locations of these brain regions were chosen to vary in depth and location across the whole brain. To meet this aim we selected the 20 deepest sources and the 20 most superficial sources from the mesh. To ensure the sources varied in position around the brain, we ensured that no more than 1 source from each AAL atlas region^52^ was chosen (Fig. 3).

We also added 3 levels of gain error to the simulated data. The gain errors were assumed to unrelated for each sensor and drawn from a normal distribution of mean 0 and standard deviation 0% (zero gain error), 2.5% and 10% of the nominal gain. To limit computation time, the exploration of gain errors was examined at a single level of spatial sampling (~ 30 mm) with 30 repetitions.

In summary, the simulated data consisted of 6000 field topographies simulated from 40 different brain regions, for 50 different levels of spatial sampling, at 3 different SNRs for both on and off-scalp systems.

### Source estimation, model comparison and spatial discrimination

#### Source estimation

Here we define a metric of spatial discrimination as the Euclidian distance at which we can confidently (p < 0.05) distinguish between the magnetic field patterns generated by two sources on the cortical mesh. We note that we could use other measures of distances such as geodesic distance. However, this may result in situations where one appears to be able to discriminate at short geodesic distances (high curvature area) but not at larger ones (either side of gyri where lead fields will be similar). Using Euclidean distance should therefore lead to a more conservative estimate of the minimum distance at which sources can be discriminated**.**

For estimating the sources we use an Empirical Bayesian Beamformer^53^ as implemented in SPM12 (https://www.fil.ion.ucl.ac.uk/spm/). This method has been used in a similar context with real data to distinguish between models of cortical anatomy^54^ and models with missing or displaced hippocampal anatomy^55^. The advantage of this method is that gives us a model evidence (free energy) value to judge the quality of the data fit (and so can be used with real or simulated data). We note that there is a significant interaction between the choice of inversion method (or assumptions) and how the data are simulated (smoothness, correlation between sources, level of correlated interference etc.). Here we explicitly simulate data that conforms to the assumptions of the inversion method so as to factor out the choice of inversion method in the following analyses. It is also therefore a best-case scenario.

#### Model comparison and spatial discrimination

Here we use Bayesian model comparison^56^ to probabilistically formalise a metric of spatial discrimination. In each case we compare a base and a target model. The base model contains the source and all neighbouring potential sources (within a certain radius), the target model is identical to the source model but with a void (excluded sources) around the true source location. This void can be made progressively larger to establish the ability of distant sources to explain the data. It is possible to explain the MEG data using both models, however the current distribution on the target model must always be more complex or less accurate than the current estimate on the base model (as the generator source is not present). The increase in complexity and/or decrease in accuracy is quantified in the model evidence of the solution (as approximated by free energy). The free energy difference between any two models is how likely (on log scale) one model is over the other. Differences in log model evidence (or free energy) of greater than 3 suggest that one model is 20 times more likely than the other. The idea is to increase the radius of the void until the explanation of the data provided by the target model is significantly (20 times) less likely than that provided by the base model. This radius of the void at which we get a significant difference between models is our metric of spatial discrimination.

Here we begin with a ring of void radius 3 mm. We increase this distance within which vertices are excluded (4, 5, 6 mm etc.) from around the true source. We keep doing this until we reach a point where we can confidently discriminate the models. The target model that is 20 times (or log difference of 3) less likely than the base model defines the spatial discrimination of a given array. This process is then repeated for each level of spatial sampling (10–60 mm in 1 mm steps) for 3 levels of signal amplitude (1, 10 and 100 nAm). This process is repeated 40 times for different brain regions in the mesh. The final results are then averaged across brain regions to obtain an average relationship between spatial discrimination and spatial sampling as a function of signal amplitude.

**Figure 1:**
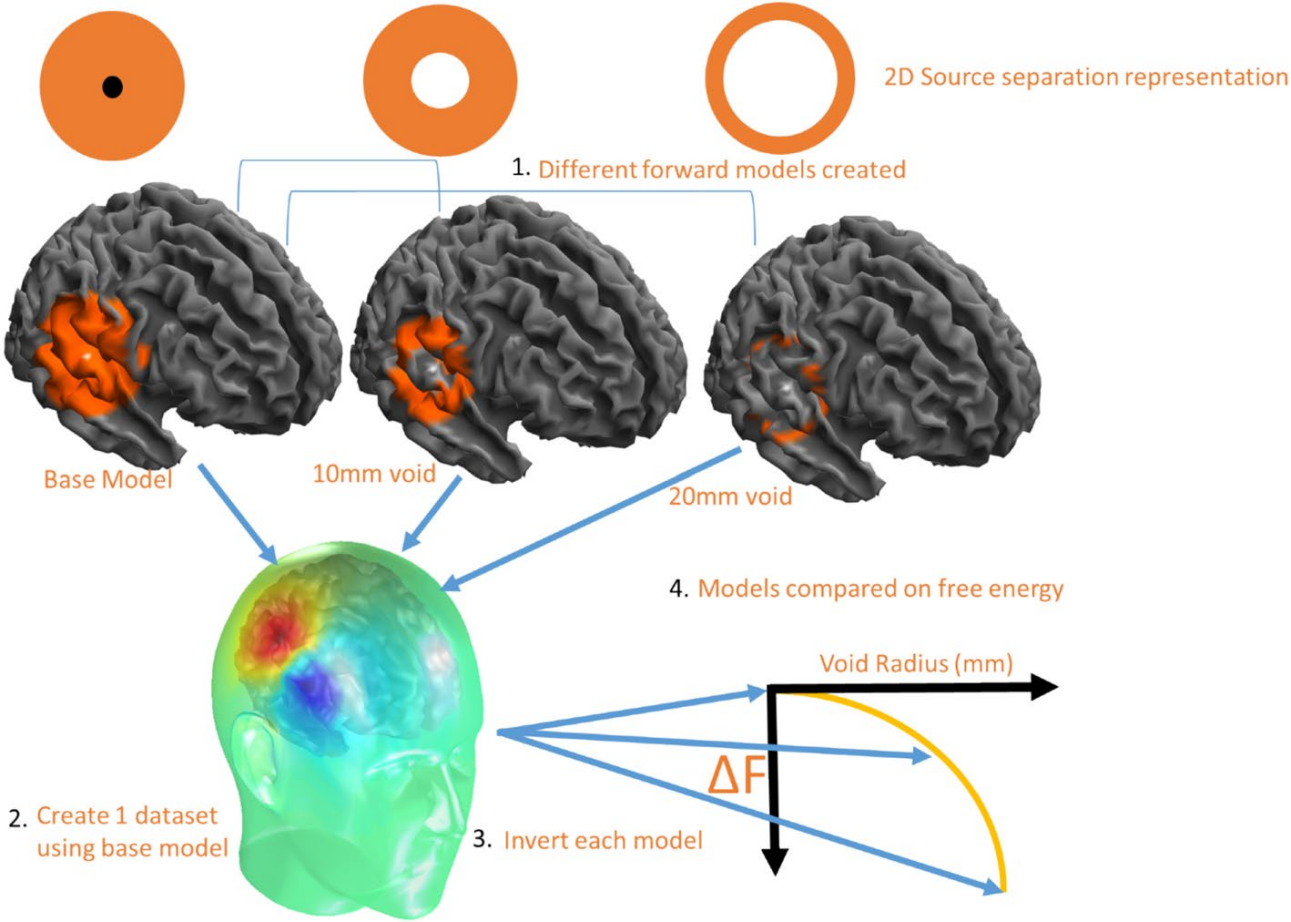
Model comparison and spatial discrimination. Different forward (or target) models are created that map to brain regions that are progressively more distant to the true generator source (at centre of circle). A 2D representation is provided with the source in black and the candidate models in orange (the contents of the lead field matrix). We then reconstruct the same MEG data onto all cortical models. Each fit has an associated model evidence or free energy value. As the void radius (excluded sources) increases, the models provide increasingly less likely (as quantified by log Free energy) explanations of the MEG data. The void radius (in mm) at which the target model is 20 times less likely (log $\Delta$ F =  − 3) is a measure of the spatial discrimination.

Practically, when we implement this approach, we do not invert the data on to the entire cortical mesh. Instead we invert the data onto a limited source space which includes the true sources and all sources within 20 mm of the true sources. This results, on average, in 420 potential sources for each base model. This model is compared successively against models that have sources within X mm (3, 4, 5… 20 mm) excluded. These models effectively form rings around the true source (see Fig. 1). This is primarily to reduce computation time. It also makes the results slightly more conservative (the spatial discrimination is slightly better if more cortex is included in both models). The explicit steps in the approach can be broken down as follows:

(1) Different generative models of the same data are proposed. The “base model” includes a source and all vertices within 20 mm of the source (top-left panel Fig. 1). All other models do not include the source (or the lead-field elements mapping sensors to the simulated location) or any potential sources (or lead-field mappings) within a void surrounding the true source (see Fig. 1). The void radius is gradually increased in 1 mm steps up to 20 mm (top panels Fig. 1). (2) The base model is used to generate a single dataset. (3) Each candidate model is inverted (using the limited source space, 20 mm radius) to explain the source-generated dataset and a free energy (or log model evidence) value is obtained. Importantly, only the base-model includes the generating source. (4) The point at which the free energy drops to − 3 (the point at which distant vertices offer an explanation that is 20 times worse than the base model) defines the spatial discrimination of the array. Similar approaches have been used elsewhere to define the discriminability of different hippocampal^43^ and cortical mesh models^57^. A more thorough introduction to the use of free energy in source inversion is given elsewhere^58^.

### Array design

**Figure 2:**
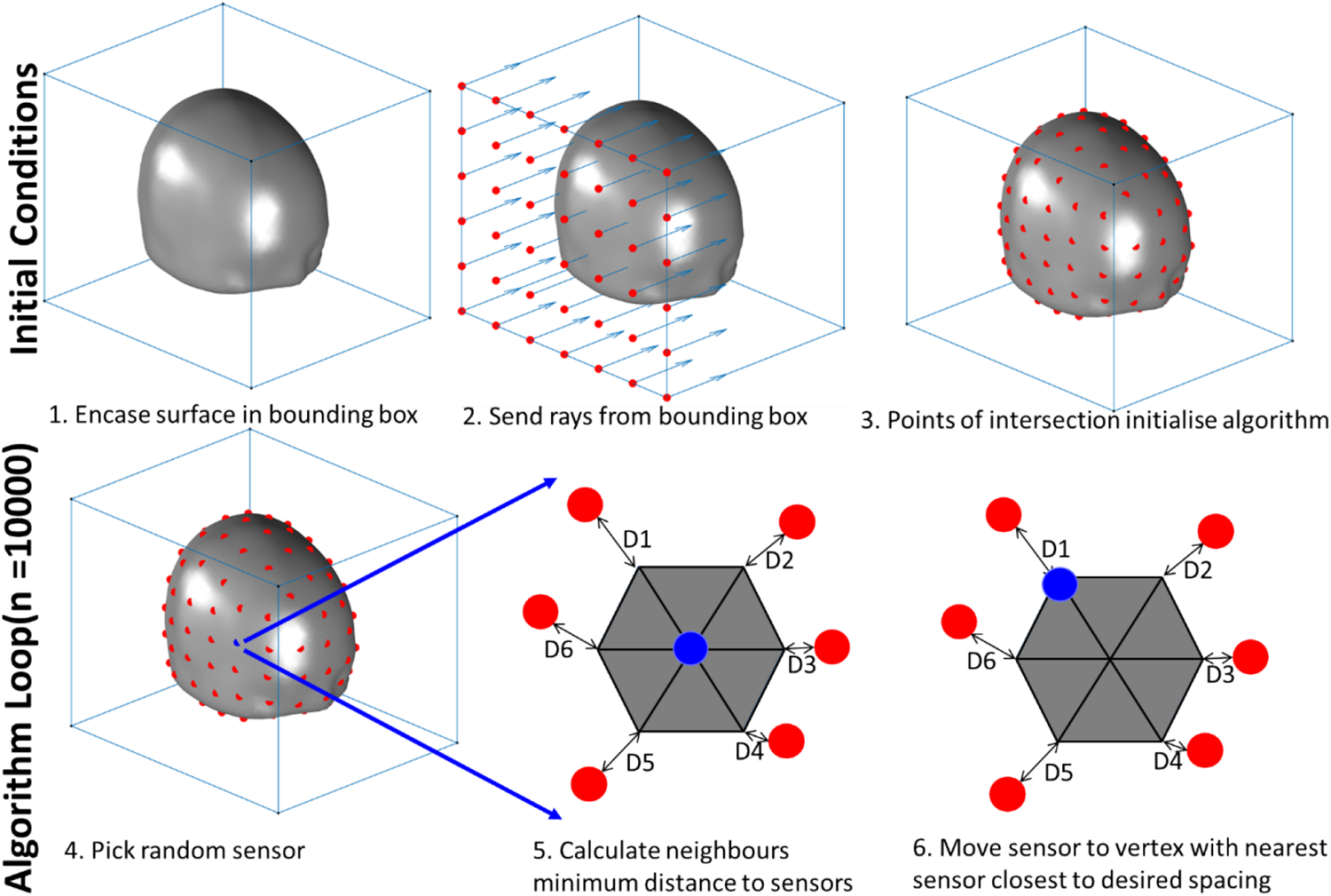
Algorithm for sensor placement. Initially the bounding box of the surface is subdivided into squares of edge length equal to the desired sampling density (panel 2). These points are then projected onto the surface to initialise the algorithm (panel 3). This is repeated for each face of the bounding box in the optimisation stage, at each loop iteration, a sensor is chosen at random (panel 4) and moved to a neighbouring vertex if the movement brings the observed sampling density closer to the target sampling denisty (panels 5, 6). This process is then repeated.

We use a point packing algorithm (Fig. 2) to position sensors on the scalp surface at increasing densities (e.g. 20, 15, 10 mm separation). There are 5 steps to the algorithm and the approach is depicted graphically in Fig. 2. (1) Initially the bounding box of the surface is subdivided into squares of equal area with an edge length equal to the desired sampling density. (2) The corners of the squares are then projected onto the surface to initialise the algorithm at approximately the correct sampling density. (3) In the optimisation stage, at each loop iteration, a sensor is chosen at random and moved to a neighbouring vertex if this brings the sampling density closer to the target sampling density (measured with Euclidean distance). (4) If there are any vertices on the surface that are farther from the nearest sensor than the target spacing, a sensor is added to that vertex. (5) This process is repeated for a large (~ 10,000) number of iterations. For the purpose of the simulations performed here the lowest sensor is positioned at the same Z coordinate (inferior-superior axis) as the most inferior part of the brain. We note an alternate approach would be to use geodesic distance instead of Euclidean distance. We choose not to as the discrepancy between the methods is only noticeable, for smooth surfaces, at large inter sensor spacing (sensor spacing >  > than radius of head). The code required to create these arrays is available via GitHub (https://github.com/tierneytim/OPM).

### Considering the effects of aliasing

Here we simulate independent Gaussian noise on each sensor. However, in reality there will be noise variance due to brain activity unrelated to the experimental manipulation or signal of interest. These sources, in particular those proximal to the sensors^59^, have the potential to introduce noise due to the aliasing of (undersampled) higher spatial frequencies. This in turn will place a limit on the effective SNR for source reconstruction. We therefore characterise the effects of aliasing when one undersamples a given topography in two ways. First, we explore how much variance a given channel count can explain in the eigenmodes derived from a high density lead field covariance (700 sensors and 20,000 brain regions separated by approximately 3 mm). These eigenmodes are obtained by singular value decomposition of the high density lead field covariance matrix. Second, we characterise how much variance a given channel count can explain in each of the 20,000 lead fields.

For a given topography an available channel count is set. By way of example we will use 100 sensors as the channel count. To initialise the algorithm we randomly sample 100 points from the set of ~ 700 potential sensor locations defined by the high density lead field. The remaining 600 empty points are filled in by nearest neighbour interpolation. This provides us with a high density “reconstructed” lead field. The squared correlation coefficient between the reconstructed lead field and the true lead field defines the variance explained. The variance unexplained is an approximation for a worst-case aliased variance. To transform this variance explained to a worst case sensor-level SNR (in a regime limited by aliasing) we can calculate it as $10{\mathrm{log}}_{10}\frac{VE}{1-VE}$. This assumes the variance explained ($VE$) is the signal and the variance unexplained is the noise ($1-VE$) and the random sensor noise is minimal.

To optimise these initial random positions a single sensor position is randomly selected. It is then moved to a neighbouring position if that move increases the variance explained. This process is repeated until the method converges on a given channel layout that maximises variance explained/minimises aliasing. The results of this method are, by their very nature, stochastic and subject to finding local minima. As such, they can be considered worst case scenarios for considering the effects of aliasing (correlated, proximal and superficial sources). We also point out that using a nearest neighbour interpolation is by no means optimal but is useful for providing an approximate estimate of the expected aliasing quickly.

### Use of experimental animals/and human participants

No experimental animals or human subjects participated in this study.

## Results

### Source locations and lead field rank

**Figure 3:**
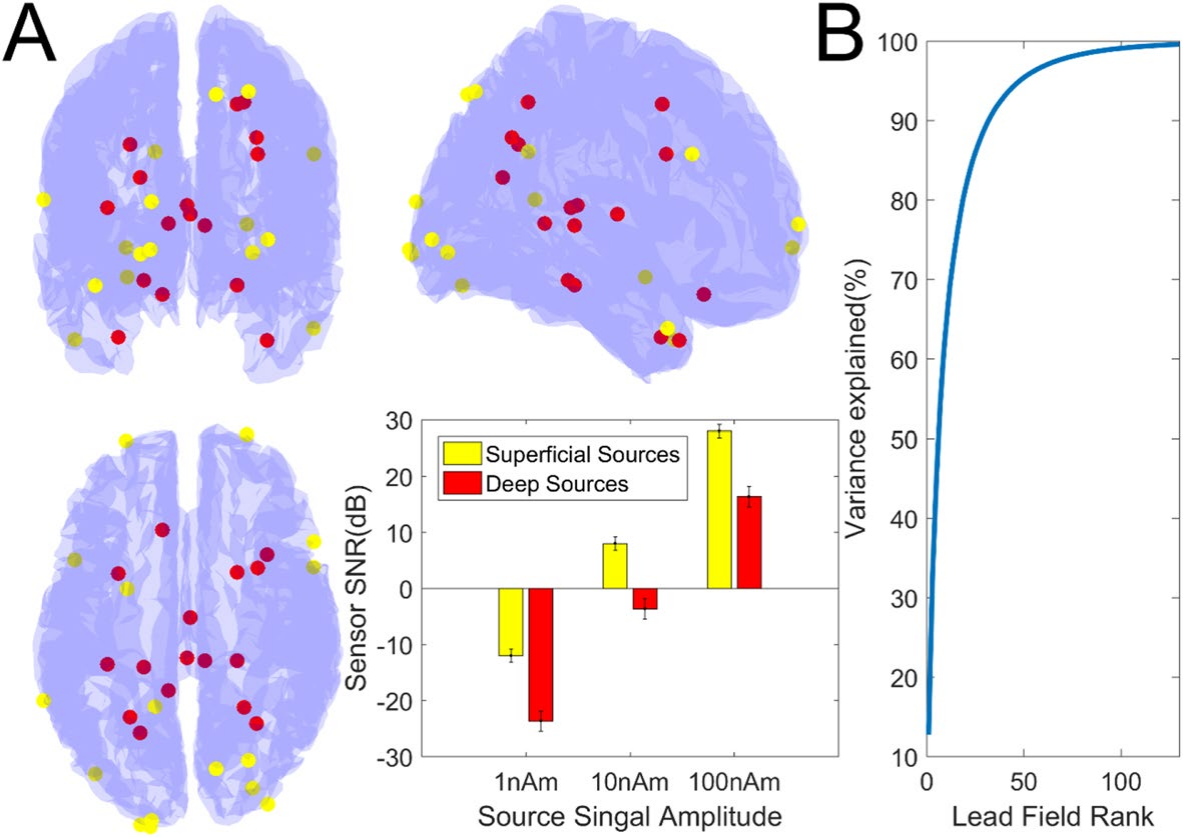
Source Locations, SNR and Lead Field Rank. In (**A**) the locations of the superficial sources are highlighted in yellow while the locations of the deep sources are highlighted in red. The resulting sensor level SNRs (in dB) obtained for our chosen signal amplitudes (1 nAm, 10 nAm and 100 nAm) are also shown (bar graph). Again, superficial and deep sources are signified in yellow and red respectively. In (**B**) we show the variance explained in our most densely sampled lead field (~ 700 sensors × 20,000 source locations) as a function of lead field rank (obtained by singular value decomposition of the lead field covariance matrix). The 95% and 99% variance explained points are approximately rank 50 and rank 100.

We first provide a descriptive account of the source models used in this study. We highlight the locations of the brain regions (Fig. 3A) used to generate the data used in these simulations (and the associated SNRs) as well as the variance explained as a function of lead-field rank (Fig. 3B) for our most densely sampled lead field (~ 700 sensors × 20,000 source locations). It is important to note that over 95% of the lead field variance can be explained with 50 spatial components and 99% of the variance can be explained with ~ 100 components. This same relationship has also been observed elsewhere^30^.

### Depth, SNR, spatial sampling and spatial discrimination: on scalp and off scalp

**Figure 4:**
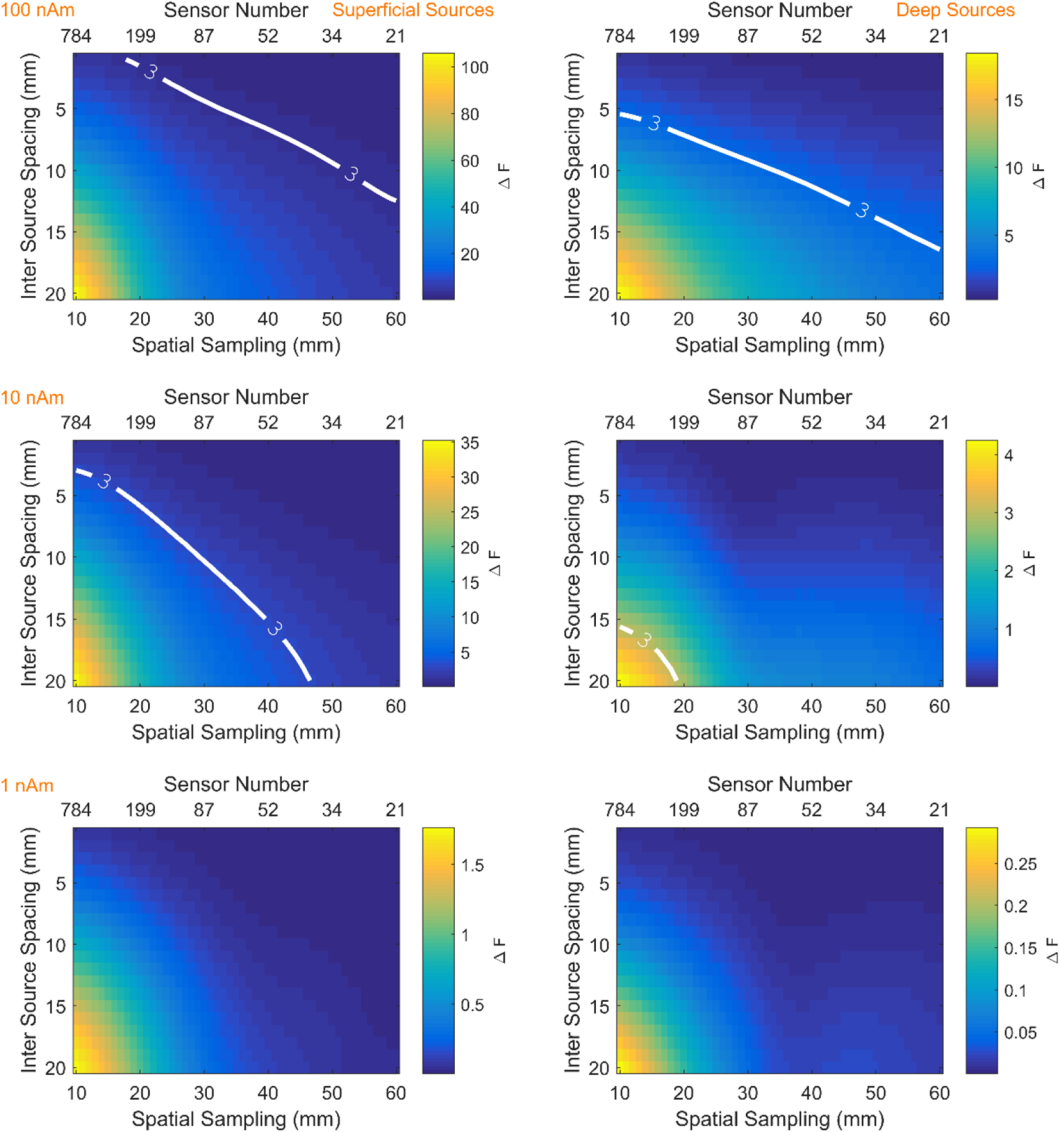
Discrimination between models as a function of spatial sampling density (or channel number). Left and right columns depict shallow and deep sources respectively. Rows show three different source amplitudes (100nAm, 10nAm, 1nAm). The colour scale shows the change in free energy relative to the base model. Thick white lines delineate the sensor spatial sampling necessary to confidently (p < 0.05 or F > 3) discriminate sources at a given inter source spacing. The respective sensor level SNRs for these figures are 28 dB, 8 dB and − 12 dB for superficial sources and 16 dB, − 4 dB and − 24 dB for deep sources. The sensors used in this simulation for point magnetometers displaced 6.5 mm from the scalp.

The results of simulations described in “Source estimation, model comparison and spatial discrimination” section are graphically represented in Fig. 4. The white contour lines indicate the point at which nearby sources can be discriminated for each candidate array. For example, for shallow sources (left column) at moderate SNR (middle row) the ability to discriminate sources at less than 5 mm, reading from the F = 3 (or p < 0.05 contour line) would require more than 200 channels (spatial sampling 20 mm). However, if one wished to discriminate sources at 10 mm (for the same moderate SNR at approximately 30 mm spacing) only 87 sensors would be required. The same curves for typical cryogenic configurations (sensors offset from the scalp by 20 mm) are shown in Supplementary Fig. S1. In this case the trends are the same except that more channels are required in order to reach the same discrimination performance.

### Number of sensors

**Figure 5:**
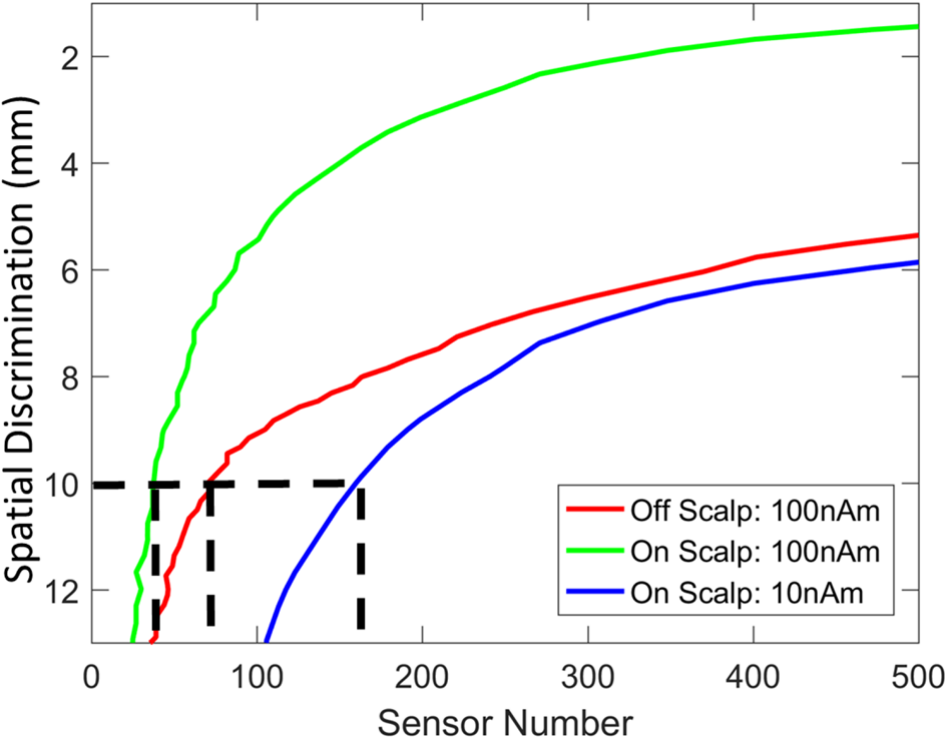
Sensor number and spatial discrimination for on- and off-scalp systems. Sensor number (x-axis) is graphed against spatial discrimination (y-axis) for both on-scalp (green, blue) and off-scalp (red) systems at different signal levels (100 nAm, 10nAm). Neither system had discrimination performance below 12 mm at 1 nAm and the off-scalp system did not reach this level at 10 nAm and so these curves are not shown (results are averaged across brain regions). While both systems are capable of achieving spatial discrimination of sources better than 1 cm across the whole brain (black dotted lines), the on-scalp system (blue and green curves) can achieve this with considerably fewer channels. Importantly, one can see for a given spatial discrimination an on-scalp system requires fewer sensors than an off-scalp system for a given signal level. The sensor to scalp distances for on and off-scalp systems are 6.5 and 20 mm respectively.

We can graph the relationship between spatial discrimination and spatial sampling for on-scalp and off-scalp systems (displaced by 20 mm) at a given SNR (Fig. 5). Consistent with previous work^50^, we found that to achieve the same spatial discrimination on-scalp systems require fewer sensors than their off-scalp counterparts for a given signal level. For example, to achieve 10 mm spatial discrimination between sources, ~ 40 rather than ~ 70 channels are required for on-scalp vs. off-scalp sensors respectively at high SNR, while ~ 150 on scalp sensors are required for moderate SNR in comparison to more than 500 sensors for the off-scalp system. Interestingly for on-scalp systems (10,100 nAm conditions), we note that beyond 100 sensors, at least 50 sensors are required for every 1 mm improvement in discrimination. The purpose of this paper is not necessarily to highlight how good an on-scalp system could be (if one had infinite sensors) but to highlight the trade off in sensor number and discriminability, which is a relatively modest return (~ 50 sensors per mm improvement) after 100 sensors.

### The impact of gain errors on spatial discrimination

**Figure 6:**
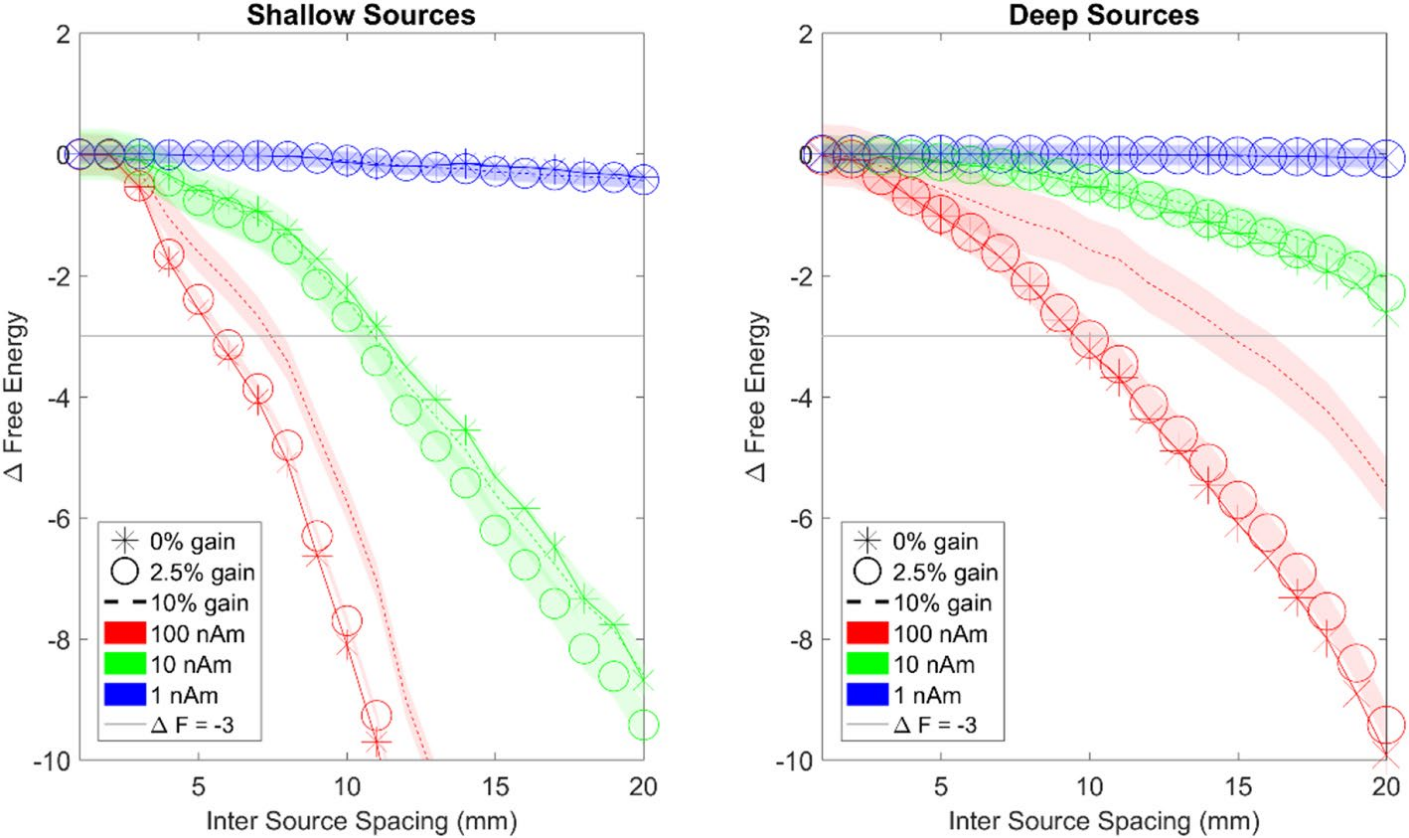
The relationship between gain errors and spatial discrimination of OP-MEG systems for shallow (left panel) and deep sources (right panel). The x axis is inter-source spacing and the y axis shows change in free energy. The point at which the curves cross the F =  − 3 line shows the distance at which the true and competing source models can be confidently distinguished. High, moderate and low SNR are signified by red green and blue curves, respectively. The thickness represents standard error. Stars represent 0% gain error, circles represent 2.5% gain error and dotted lines represent 10% gain error. The horizontal light grey lines represent the free energy threshold of − 3 that defines the spatial discrimination of the array. The same symbols and colour schemes are used for the right panel to describe deep sources. Noticeable differences in discrimination due to gain errors are only discernible at high SNR.

From the previous section we can conclude that there was only a modest return as the number of scalp sensors increased beyond 100. In this section, we fix the sensor number at 100 and examine the effect of calibration or gain errors on spatial discrimination. The simulations include 30 repetitions of 3 different gain errors (0%, 2.5% and 10%) for 3 different signal levels (1 nAm, 10 nAm, 100 nAm with 100 fT standard deviation sensor level noise). Figure 6 shows the spatial discrimination that can be achieved for different levels of gain errors. It can be seen that the gain errors have the biggest impact at high SNR. Note the divergence between the 0% (stars) and 10% (dotted) gain error curves for 100 nAm (red). Interestingly the gain errors appear more detrimental for deeper sources. For deeper sources (right panel) at high SNR (red curves) achievable discrimination degrades from 10 to 16 mm at 10% gain error. At moderate and low levels of SNR the effects of gain errors are masked by the noise. These gain errors would be analogous to random sensor orientation errors of ~ 0, 13, and 26 degrees respectively when the field on the other axes is small (assuming a cosinusoidal dependence between orientation errors and gain errors, e.g. acos (0.975) × 180/pi =  ~ 13 degrees).

### The effects of aliasing

**Figure 7:**
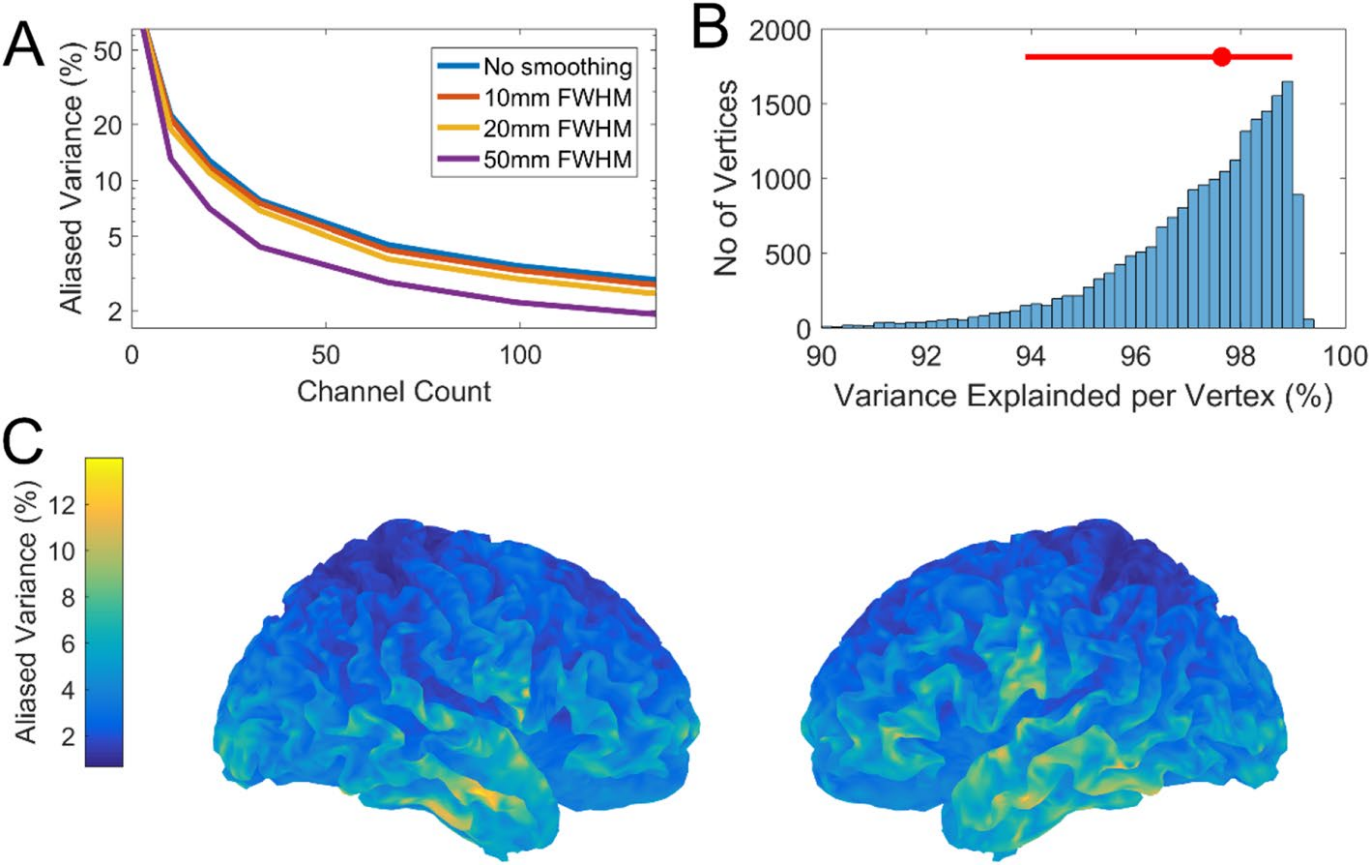
The effects of aliasing. In (**A**) the percentage of aliased variance (from the lead field covariance matrix) is plotted as function of channel count. The Y axis is logarithmically scaled. This is repeated for different levels of source space smoothness (no smoothing, 10 mm, 20 mm and 50 mm). In (**B**) the histogram of variance explained across the entire cortex is calculated for an array of 100 sensors. The median variance explained (red dot) is highlighted as well as the 2.5% and 97.5% quantiles (red bar). In (**C**) this same information is visualised across the cortical surface to characterise the spatial profile of aliasing.

We have characterised the relationship between spatial sampling and spatial discrimination as a function of SNR to highlight a trade-off between spatial discrimination and channel count. This trade-off can lead to situations where brain activity is under-sampled and therefore aliases. This aliasing will manifest as noise that places an effective limit on the SNR. In Fig. 7A we can see that aliasing reaches approximately 4–5% for between 60 and 100 sensors for point sources. For cortical generators with some spatial extent this percentage decreases marginally. The marked decrease for generators of 50 mm FWHM is interesting but perhaps not physiologically reasonable. This 4–5% aliased variance corresponds to sensor level SNR (magnitude) of between 4.4 and 4.9 (~ 13–14 dB). We can conservatively extrapolate from Fig. 4 (10 nAm, shallow source corresponding to SNR of 8 dB) that in the aliased regime spatial discrimination would be limited to approximately 10–15 mm for 50–100 sensors. In Fig. 7B we decompose the aliasing into its regional contribution for our suggested channel count of 100. We can see that the vast majority of brain regions (95% interval) can be sampled with 100 sensors to have between 6 and 1% aliasing. This same information is represented in Fig. 7C on the brain’s surface. Unsurprisingly, the areas that contribute most to the aliasing are the more superficial sources (although these are few in number).

## Discussion

We have presented a probabilistic model comparison framework to quantify spatial discrimination of competing sensor arrays. We show that spatial discrimination of 1 cm requires relatively few on-scalp sensors (< 100). This corresponds to an inter-sensor distance of approximately 3 cm. For this sensor layout, gain errors have the greatest impact discrimination at high SNR but its effects are masked at moderate or low SNR. We also explore the potential impact of noise due to aliasing on these sparse arrays and find that this could account for between 1 and 6% of signal variance.

Here we show that adequate discrimination performance for on-scalp sensors is achievable even though we are far from the theoretical limits of spatial discrimination for these sensors. Indeed recent studies^30,60^ have shown that, due to the higher spatial frequency fields now measurable, to fully exploit the potential of OPM systems one would require of the order 300 sensors. That being said, we show even when SNR is limited by a large degree of aliasing (e.g. 15% corresponding to ~ 8 dB SNR limit—Fig. 4, left column, second row) then spatial discrimination will be limited to be between 10 and 15 mm for between 50 and 100 sensors. This problem will be most acute when the sensor level noise (random noise) is small in comparison to the aliased signal. This can occur after averaging data arising from highly covariant sources or when the sensor has a much lower noise floor than the background spontaneous activity of the brain (e.g. in the 1–20 Hz band). In cases where this spontaneous activity is not correlated with experimental design this source of variability should reduce with averaging, assuming stationarity of the neural response^61^. A straightforward approach to mitigate this problem is to consider non-uniform sampling arrays specific to experimental or clinical regions of interest^59^.

We have made several idealistic assumptions. Practically, achievable discrimination performance may be bounded by other factors. For example it has been shown that forward modelling errors limit discrimination at high SNR^50,62^. Here, we assume that the source model is known and we have created and reconstructed the data under the same forward models. Other studies have looked at these models in more detail^63,64^ and we do not examine this factor directly. That said, increasing the channel number will not solve these issues. We had initially expected gain errors to have similar consequences to forward modelling errors but it appears these are much less pernicious. This is because the gain changes have been considered independent across sensors (rather than systematic for lead-field errors) and indeed we found that for practical OPM gain errors (± 2%) there were negligible effects on discrimination performance, similar to random coregistration errors^65^. However, there may be instances when gain changes have some correlated spatial structure across the array (for example as the subject moves through a magnetic field gradient) and this would most likely be more detrimental to the source modelling. We should point out that here we have focused on source reconstruction. However, some studies which operate in sensor space exclusively, for example studies using Multivariate Decoding^66^, will likely have different performance constraints.

The model comparison framework could be extended in a number of ways. For instance, it could be used to target specific brain regions or to compare other wearable brain imaging systems such as EEG. A more thorough comparison of EEG and on-scalp sensors has been performed elsewhere^29,60^. There are other ways to define spatial discrimination such as low correlation between sources^15,50^ or examination of point-spread functions considered resolved^50^. However, defining spatial discrimination is arguably a statistical problem. The proposed statistical framework has also been used extensively to model laminar sources^44,67,68^ in MEG as well as make probabilistic statements about deep structure activity^43,69^ where spatial precision is paramount. This probabilistic nature of variational Bayes is a strong motivation for adopting such a framework for assessing spatial discrimination. While the variational framework allows for approximate Bayesian inference in an efficient and accurate way^56,70^ it is important to recognise it does so under some key assumptions. The primary assumption is that the distribution of estimated parameters is well approximated by a Gaussian around its mode. This can be broken if the true underlying distribution has a mode that is not near the majority of the probability mass (heavy tailed distribution or multimodal distributions). In practice however it can be shown that in a large data limit this is of limited concern^71^.

We have provided a simulation-based framework for users of OPMs to establish how many sensors are required for neuroscientific applications to achieve a desired spatial discrimination. We find adequate discrimination (< 1 cm) can be obtained with relatively few sensors (< 100) at coarse spatial samplings (> 30 mm) at high SNR for both deep and superficial sources. Beyond this point, improving discrimination comes at a cost of nearly 50 sensors per mm improvement. Furthermore, aliasing due to undersampling (which is correlated with the experimental design) may place an effective limit on the SNR available for spatial discrimination. For between 50 and 100 sensors we expect this aliasing to typically be between 2 and 6% and rarely exceeding 10%. This limits the achievable discrimination to between 5 and 15 mm spatial discrimination for these channel counts. Finally, we also demonstrate that the dominant determinant of spatial discrimination in an OPM system is not gain changes/calibration errors but the magnitude of the physiological response and the depth of the source.

## Supplementary information

Supplementary Information
