# Supplementary material for: Pragmatic spatial sampling for wearable MEG arrays
Source: Sci Rep. 2020 Dec 10;10:21609. doi: 10.1038/s41598-020-77589-8 (PMC7729945; doi:10.1038/s41598-020-77589-8)
Supplement: Supplementary file 1 — Supplementary Information [file 41598_2020_77589_MOESM1_ESM.docx]

**Pragmatic spatial sampling for wearable MEG arrays**

Tim M Tierney^1*^, Stephanie Mellor^1^, George C O’Neill^1^, Niall Holmes^2^, Elena Boto^2^, Gillian Roberts^2^, Ryan M Hill^2^, James Leggett^2^, Richard Bowtell^2^, Matthew J Brookes^2^, Gareth R Barnes^1^

1. Wellcome Centre for Human Neuroimaging, UCL Institute of Neurology, London, UK, WC1N 3BG
2. Sir Peter Mansfield Imaging Centre, School of Physics and Astronomy, University of Nottingham, University Park, Nottingham, UK, NG7 2RD

*Correspondence: tim.tierney.12@ucl.ac.uk


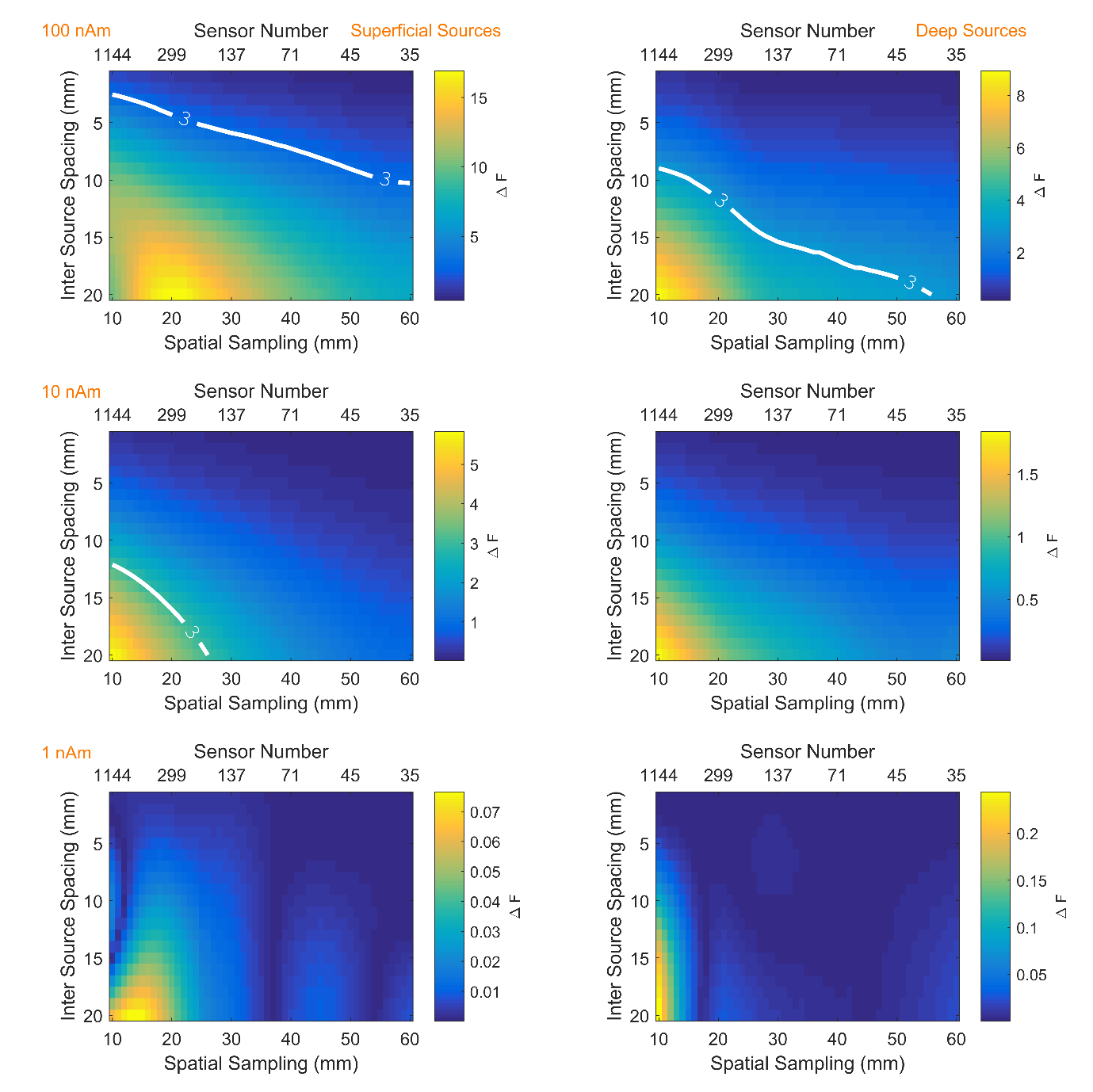


Figure S1. Discrimination between models as a function of spatial sampling density (or channel number). Left and right columns depict shallow and deep sources respectively. Rows show three different source amplitudes (100nAm, 10nAm, 1nAm). The colour scale shows the change in free energy relative to the base model. The thick white line (Free Energy contour) delineates the sensor spatial sampling necessary to confidently (p<0.05 or F>3) discriminate sources at a given inter source spacing. These results are obtained by averaging across the brain regions used to generate the simulated data. For lower SNR and sparse sampling the Free energy is not quite monotonic. However, these local maxima are statistically uninteresting (as they are of the order of .1 log units and do not constitute a significant difference between models (where 3 units would be our normal threshold).The sensors used in this simulation for point magnetometers displaced 20 mm from the scalp.
